# Potential immunomodulatory effects of the extract from *Artemisia frigida* Willd on loaches infested with *Aeromonas hydrophila* revealed by microRNA analysis

**DOI:** 10.3389/fgene.2025.1584539

**Published:** 2025-04-15

**Authors:** Yue Zhao, Yuqing Qiu, Lishang Dai, Hong Wang

**Affiliations:** ^1^ School of Biological and Environmental Engineering, Chaohu University, Hefei, China; ^2^ School of Traditional Chinese Medicine, Wenzhou Medical University, Wenzhou, China; ^3^ Chaohu Regional Collaborative Technology Service Center for Rural Revitalization, Chaohu University, Hefei, China

**Keywords:** microRNA, *Artemisia frigida* Willd, loach, *Aeromonas hydrophila* infection, immune regulation

## Abstract

*Artemisia frigida* Willd is the most widely distributed *Artemisia* plant in the steppe and has a long history of medicinal applications in folk, especially as Mongolian medicine. Modern pharmacological research shows it exhibites biological activities such as antioxidant, anti-inflammatory and antibacterial. However, antibacterial applications of *A. frigida* in fish have not been reported. Loach is a kind of small economic fish with delicious meat and high nutritional value, which has high market value and demand in China. Nowadays, loach aquaculture technology is more mature, but the effective prevention and control of bacterial infectious disease outbreaks still need to be solved, for example, infection with *Aeromonas hydrophila* can cause high prevalence and mass deaths, leading to huge economic losses. MicroRNAs (miRNAs) regulate many biological processes, including an important regulatory role in the antibacterial immune response in fish, and immune-associated miRNAs have now been identified in a wide range of fish species, but less research has been carried out on loach miRNAs. To identify miRNAs related to antibacterial immunity in loach and to understand the potential immunomodulatory mechanism of *A. frigida*, we infected both *Artemisia*-fed and non-*Artemia*-fed loaches with *Aeromonas hydrophila*, and then constructed two small RNA libraries using high-throughput sequencing technology. Bioinformatics analysis identified 924 and 923 conserved miRNAs in control and AF (*Artemisia frigida*) treated samples, respectively, and 30 (26 upregulated and 4 downregulated) differentially expressed miRNAs were screened. Six immune-related miRNAs were selected for fluorescence quantitative PCR used to verify the accuracy of the sequencing results. Further target gene prediction and functional analysis of 30 differential miRNAs showed that the target genes of these miRNAs were involved in the regulation of several innate and antibacterial immunity-related pathways, including endocytosis, apoptosis, phosphatidylinositol signaling system, RLR signaling pathway, TLR signaling pathway and NLR signaling pathway. This study helps to deepen the understanding of the mechanism of miRNA regulation of antibacterial immune response in loach, and provides new insights into the application of the Chinese herb *A. frigida* in fish.

## 1 Introduction

The loach (*Misgurnus anguillicaudatus*) is a small-sized fish species of warm water, mainly inhabited the bottom of lakes, ponds and other shallow water areas (Wang et al., 2018). The muscle of the loach is rich in high quality proteins and fatty acids, with high nutritional value, and the meat is tasty, so it is very popular among the public (Yang et al., 2023). In China, loach has become one of the most primary economically cultured fish with high market value and demand. However, with the rapid advancement of loach aquaculture, it is also facing more and more challenges, and one of the biggest threats is the outbreak of infectious diseases (Ofek et al., 2023). Fish diseases raised by infectious organisms such as bacteria, viruses and parasites in water usually have high death and morbidity rates, with pathogenic bacteria being the chief cause of infectious diseases (Ahmed et al., 2022; Sarkar et al., 2021). It is universally acknowledged that *Aeromonas hydrophila* is a Gram-negative bacterium of high virulent that is ubiquitous in aquatic ecosystems (Mzula et al., 2019). As one of the main causative organisms in freshwater fish aquaculture, infection by *A. hydrophila* can lead to Motile *Aeromonas* Septicemia (MAS), which usually manifests symptoms such as gastrointestinal haemorrhage, skin ulceration, ascites and gill stasis (Abdelhamed et al., 2017; Abdul Kari et al., 2022). The highly contagious nature of the disease can eventually lead to mass mortality of cultured species, causing huge economic losses (Loch et al., 2017). As a result, the search for safe and effective bactericidal drugs and the elucidation of related antimicrobial mechanisms are of serious significance for the healthy development of loach aquaculture in the future.

As high-throughput sequencing technology improves by leaps and bounds in recent years, in which deep sequencing of miRNA has been extensively implemented in animal and plant research. MicroRNAs, abbreviated as miRNAs, are a class of endogenous single-stranded small molecule RNAs that can downregulate gene expression at the post-transcriptional level, with a length of about 19–24 nt (Huntzinger and Izaurralde, 2011; Stern-Ginossar et al., 2007). People have a clear understanding of the biosynthetic pathway of animal miRNA at present. First, the gene encoding miRNA in the nucleus is transcribed into the primary transcription product pri-miRNA by RNA polymerase II. Subsequently, Drosha enzyme, an RNaseIII endonuclease, *cleave* it into a hairpin precursor miRNA (pre-miRNA), characterized by a 2-nucleotide protrusion at the 3′ end (Gregory et al., 2004; Kloosterman and Plasterk, 2006). The mature miRNA 3′ end is obtained in this step. Following the initial shearing in the nucleus, pre-miRNA is exported to the cytoplasm via transporter, where it is further cleaved by Dicer, another RNase III endonuclease, to form a double-stranded miRNA intermediates (miRNA: miRNA*) (Hutvágner et al., 2001). In the end, the mature miRNA strand of the double strand binds to the RNA-induced silencing complex (RISC) and the other complementary strand is degraded in the cytoplasm. Mature miRNAs regulate gene expression as demonstrated by the miRNA binding to the 3′ untranslated region (UTR) or coding region of the target mRNA through base pairing, thereby directing RISC to degrade the mRNA or inhibit translation (Schnall-Levin et al., 2011). In animals, the regulation of gene expression by miRNAs mostly involves repressing the translation of transcription products, while the stability of mRNA transcripts is not affected. Nowadays, the identification and functional analysis of miRNAs have become increasingly prominent in the aquaculture industry. Studies have shown that a large number of miRNAs have been identified in multiple fish species, and these miRNAs are related to biological processes such as organ formation, growth and development, osmotic and immune regulation (Kate et al., 2023; Zhao et al., 2020). Interestingly, most people spotlight on the research of immune-related miRNAs in fish. For example, miR-144-5p was found to downregulate the expression level of the target Hsp90α in experiments with Japanese flounder infected with *Edwardsiella tarda*, embracing a reduction in the expression of inflammation-related cytokines thereby promoting bacterial infection (Li et al., 2023). Zheng et al. also found that miR-144-3p promotes the immune escape of *Vibrio anguillarum* and *V. harveyi* in fish by inhibiting the expression of inflammatory cytokines (Zheng et al., 2023). Zhou et al. reported that many miRNAs were engaged in innate immune-related pathways in the *Siniperca chuatsi* spleen of ISKNV infection (Zhou et al., 2022a).

*Artemisia frigida* Willd. (*A. frigida*) is a perennial herbaceous plant in the genus *Artemisia* of the family Compositae, mainly distributed in southwest and northwest of China (Tian et al., 2022a). The aerial parts of *A. frigida* are harvested in autumn for medicinal purposes, and are commonly used in Mongolian traditional medicine to treat arthritis, rheumatoid, damp-heat jaundice and other diseases (Borchuluun et al., 2021). Modern research has shown that flavonoids, steroids, phenylpropanoids and phenolic acid components are the main active ingredients of *A. frigida*, which have antibacterial and antioxidant (Olennikov et al., 2019), anti-inflammatory (Wang et al., 2016), anticancer (Hussain et al., 2024) and other biological effects. At present, the research on *A. frigida* is mainly focused on phytochemistry, quality control, cultivation and breeding (Ma et al., 2019a; Liu et al., 2016; Tian et al., 2022b), and its application in fish has not been reported. Active compounds with antibacterial properties in the extract from *A. frigida*, such as flavonoids and terpenoids, could have antioxidant and immunomodulatory effects on fish (Talib and Mahasneh, 2010). In addition, the use of phytobiotics in loach culture is safer, more environmentally friendly and more economical than traditional fish antibiotics (Ma et al., 2019b). miRNAs are important regulators of the body’s immune response and undergo changes in inflammation mediated by a wide range of bacterial infections, but how bacteria regulate host miRNA is still unclear. To preliminarily understand the mechanism of miRNA in the immune regulation of *A. frigida*, we studied the miRNAs and mRNAs profiles of loach of *Aeromonas hydrophila* infection after treatment with *A. frigida*. In this paper, we aim at deepening the understanding of the role of miRNAs in loach, provide a new insight for the healthy and sustainable development of the loach aquaculture, and furnish new ideas for the innovative application of *A. frigida*.

## 2 Materials and methods

### 2.1 Plant material

The aerial parts of *A. frigida* were originally collected from Miyi county, Sichuang Province, China, in July 2022, and identified by one of the authors, Xudong Zhou. A voucher sample (specimen No. LH2022207) was deposited at the Herbarium of Hunan University of Chinese Medicine, Changsha, China. The *A. frigida* (5.0 kg) were immersed and extracted in 8 L 95% aqueous ethanol by reflux extraction for three times, each time 3 h. The solvent was evaporated under vacuum to obtain a crude extract (620 g).

### 2.2 Source of loaches

Eighty healthy loach Individuals were purchased from Wenzhou Ouhai River Fish Market (Zhejiang, China), with average initial body weight of 10 ± 0.5 g. They were stored in the research laboratory of Wenzhou Medical University and divided into two 20 L water tanks. Under the condition of room temperature 25°C and 12 h of light, water was changed twice daily and commercial feed for the loach was fed after the water was changed. Loaches were acclimated in the laboratory for 1 week before the formal experiment.

### 2.3 Trial design and sample collection

The feed was treated before the start of the experiment and feed were mixed with the extract from *Artemisia frigida* at 100:1. Loaches were randomly divided into the control and the AF (*Artemisia frigida*) treatment groups with 40 loaches of each. The AF group was fed 5 g of feed mixture with the extract, and the control group was fed an equal amount of normal feed. Bacterial challenge test was performed 3 h later. *Aeromonas hydrophila* suspension was added to the control group and the AF group to maintain the bacterial concentration in the water at 1 × 10^5^ colony forming units (CFU) mL^−1^. All loach rearing conditions were kept the same as before the experiment. Liver tissues were collected from the control and the AF groups at 3, 6, 12, 24, 48, and 72 h post-infection, respectively, and each sample consisted of three loaches. The loach was dissected and collected tissues after the freezing halo on the ice, immediately plunged into liquid nitrogen for 10 min, and then stored to −80°C until total RNA was extracted for miRNA sequencing and validation.

### 2.4 RNA extraction, miRNA library construction and miRNA-seq

Total RNA from liver tissue of each sample was prepared using Trizol reagent. The total RNA concentration and purity were assessed using a Thermo Scientific NanoDrop 2000 (Waltham, Massachusetts, United States), and integrity was identified by RNA-specific agarose electrophoresis. Two isolated small RNA libraries were constructed accurately according to the instructions of NEB Next Multiplex Small RNA Library Prep Set for Illumina (Ipswich, Massachusetts, United States) kit. Briefly, the 5′ and 3′ adaptors of 1 μg total RNA were ligated by Ligation Enzyme Mix, and then the RNA was reverse transcribed into double-stranded cDNA by Superscript II reverse transcriptase, followed by PCR amplification. After the PCR product was separated by 15% PAGE gel, the library was finally obtained. Small RNA libraries were analyzed for QC and average size of inserts was approximately 140–150 bp. The sequenced libraries were quantified on a Bioanalyzer 2100 system (Agilent) using the Agilent High Sensitivity DNA Assay and subsequently handed over to Shanghai Personal Biotechnology Cp. Ltd. for deep sequencing on the NovaSeq 6000 platform (Illumina).

### 2.5 Bioinformatics analysis of sequencing data

The raw data is first filtered using the Personalbio company’s self-developed script to obtain clean data by removing sequences containing splices, sequences containing poly - N, and low-quality sequences. Clean reads with lengths from 1 nt to 36 nt are filtered and deduplication is performed to obtain unique reads for subsequent analysis. Subsequently, unique reads were mapped to the piRBase database and Repbase database to filter out piRNAs and repetitive sequences. Then the clean reads were compared with the Rfam database, from which some non-coding RNAs (ncRNAs) were annotated, including rRNA, tRNA, snRNA, snoRNA, etc. Finally, the remaining sequences were used to screen out conserved miRNAs by comparison with known miRNAs from miRbase 22.0 (http://www.mirbase.org/). Because the measured species did not have a reference genome, no novel miRNA prediction analysis was performed.

### 2.6 Differential expression analysis of miRNAs

Based on the number of sequences compared with mature miRNAs, the Reads Count value of the measured miRNAs was counted using the featureCounts software, and the first abundance in the homonymous miRNA was taken as the expression abundance of the miRNA. In order to know the miRNAs with significant changes in expression between the control and AF treatment groups, DESeq (version1.39.0) software was conducted to analyze the difference of miRNA expression, transcripts with |log2FoldChange| > 1 and P-value <0.05 were considered as differentially expressed miRNAs (DEMs).

### 2.7 Real-time quantitative PCR of miRNA

Six randomly selected immune-related DEMs were analysed by real-time fluorescence quantitative PCR, which was used to verify the reliability of miRNA-seq results. Based on the mature miRNA sequences obtained by sequencing, Primer 5 software was used to design the miRNA upstream primers, and universal primers were used for the downstream primers. Total RNA of the two groups of liver tissues prepared above was used as a template for cDNA synthesis. The miRNA 1st Strand cDNA Synthesis Kit (by tailing A) (Vazyme, Nanjing, China) was used to reverse transcribe 1 μL of total RNA to cDNA according to the manufacturer’s description. The cDNA obtained from the reaction was immediately used for quantitative fluorescence analysis. The 20 μL reaction system was configured according to the protocol of the *TransStart®* Top Green qPCR SuperMix (+DyeⅠ) kit, including 1 μL of cDNA, 0.5 μL each of specific and universal primers, 10 μL of SuperMix, and 8 μL of nuclease-free water. Then the qPCR conditions were set on *QuantStudio™* Real-Time PCR System (ABI, United States): 94°C for 30 s, 94°C for 5 s, annealing 55°C for 15 s, 72°C for 10 s, 40 cycles. Start the program for real-time fluorescence quantitative PCR. The results were calculated by 2^−ΔΔCt^ method, and the expression of miRNA was standardized with β-actin as the internal reference. The experimental operation of each miRNA was performed in triplicate, and all sequences of primers are shown in Table 1.

**TABLE 1:** Primers sequence for qRT-PCR.

miRNA	Mature sequence	Forward primers(5′→3′)
manu-mir-1-9	UGG​AAU​GUA​AGG​AAG​UGU​GUG​A	GCG​CGT​GGA​ATG​TAA​GGA​AGT
manu-mir-202-3	UUC​CUA​UGC​AUA​UAC​UUC​UUU​G	CGC​GCG​TTC​CTA​TGC​ATA​TAC​T
manu-mir-27-3	UUC​ACA​GUG​GCU​AAG​UUC​UUC	GCG​CGT​TCA​CAG​TGG​CTA​AG
manu-undef-374	UUC​ACA​GUG​GCU​AAG​UUC​AGU	GCG​CGT​TCA​CAG​TGG​CTA​AG
manu-undef-508	UAG​UUU​GAU​ACA​CAG​CAC​AAG	CGC​GCG​TAG​TTT​GAT​ACA​CAG
manu-undef-411	UUA​CAA​UUA​AAG​GAU​AUU​UCU​U	CGC​GCG​TTA​CAA​TTA​AAG​GAT​A
β-actin		AGA​GAG​AAA​TTG​TCC​GTG​AC

### 2.8 miRNA targets prediction and enrichment analysis

To investigate the potential antimicrobial mechanism of DEMs after *Artemisia frigida* treatment, these miRNAs were analysed for the prediction of target genes. The 3′UTR sequence of the messenger RNA of the species was used as the target sequence for comprehensive target gene prediction using MiRanda (v3.3a). GO (Gene Ontology) enrichment analysis was performed on all predicted miRNA target genes using topGO (v2.50.0) to determine the major biological functions they perform. Additionally, target genes of differential miRNAs, were used to KEGG (Kyoto Encyclopedia of Genes) enrichment analysis using clusterProfiler (v4.6.0) software for obtaining the metabolic pathways and signaling pathways that these target genes are mainly involved in. The significant enrichment pathway with p-value less than 0.05 was mainly concerned.

## 3 Results and discussion

### 3.1 Analysis of small RNA-seq data

**FIGURE 1:**
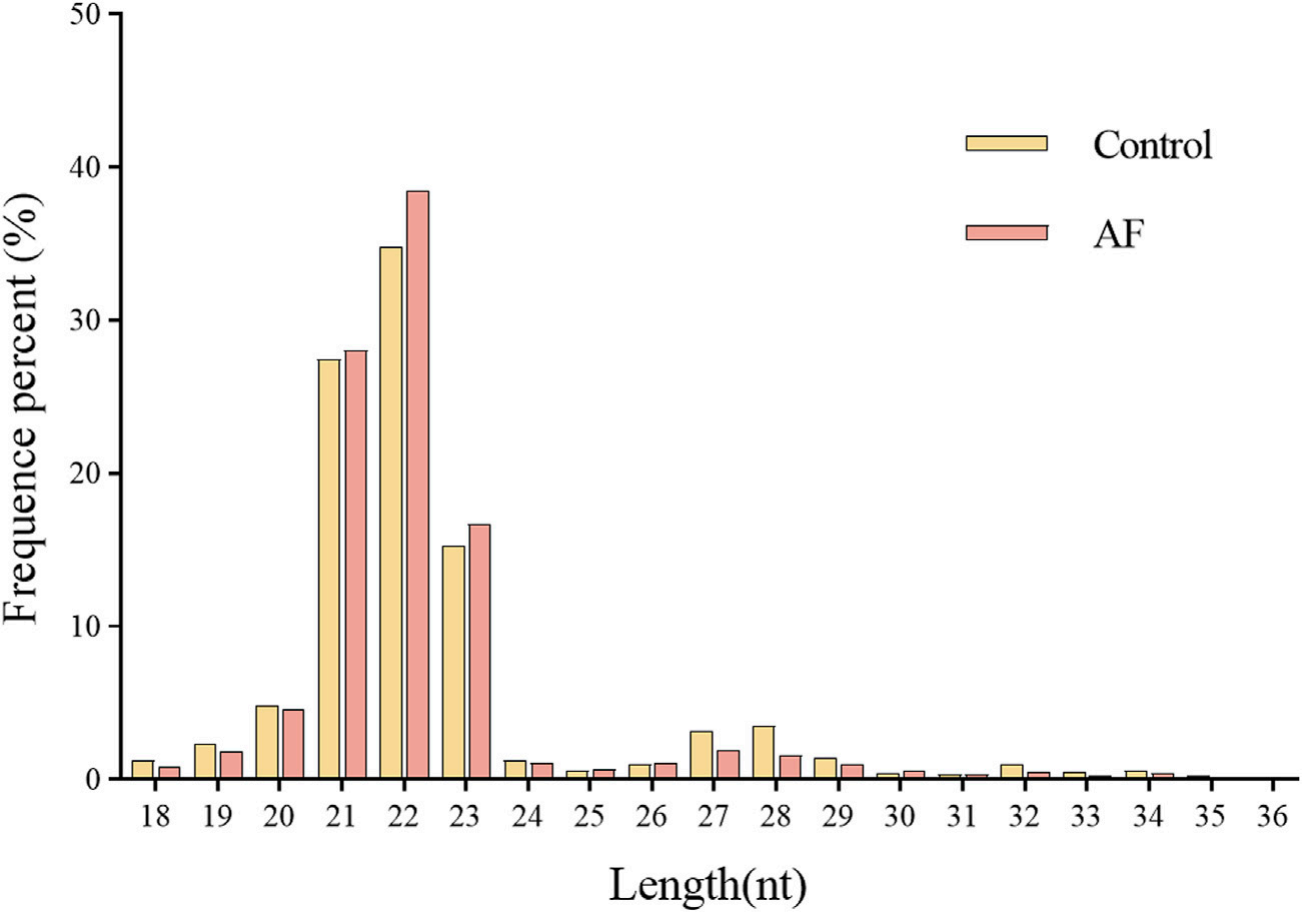
The size distribution of clean reads obtained from the Illumina sequencing of loach small RNAs libraries.

Two miRNA libraries were constructed using Illumina platform for small RNA transcriptome analysis of loach liver tissues from control (CK) and *Artemisia frigida* (AF) groups, respectively. After statistics, 16,239,217 and 17,343,492 raw sequences were obtained from CK and AF libraries, respectively (Table 2). The raw reads were subjected to a series of filters to obtain 14,660,513 and 15,916,618 clean reads for subsequent analyses. The clean reads with lengths between 18–36 nt were counted, and the distribution of sequence lengths in the CK and AF groups was similar. A majority of the sequence lengths were clustered at 21–23 nt (Figure 1), and 22 nt was the most abundant length in both groups. The clean reads were de-duplicated, i.e., identical sequences were merged to obtain unique reads for subsequent small RNA annotation.

**TABLE 2:** Sequence statistics of high-throughput sequencing data.

	Control	AF
Raw reads	16,239,217	17,343,492
Clean reads	14,660,513	15,916,618
Clean reads%	90.28	91.78
Rfam alignment	94276	72778
miRBase alignment	14790	15576
Preserved miRNA	924	923

Clean reads%: The percentage of Clean reads count in Raw reads count.

### 3.2 Summary of small RNA annotation

The unique reads were compared to the Rfam13 database, from which four known classes of non-coding RNAs (ncRNA) were identified, including rRNAs, tRNAs, snRNAs and snoRNAs. The reads of the four ncRNAs annotated by the CK group were 72221, 4324, 2577, and 15154, respectively (Table 2; Supplementary Table S1). The AF group had fewer reads annotated to each ncRNA than the CK group, with sequence counts of 55347, 3901, 2292 and 11338, respectively. Remaining reads that did not get annotated were subsequently mapped to the miRBase database for miRNA candidate identification. After blast with the mature miRNA sequences of all animals in the database, 924 and 923 conserved miRNAs with different degrees of expression in the samples of CK and AF, respectively were screened.

### 3.3 Identification of differentially expressed miRNAs

**FIGURE 2:**
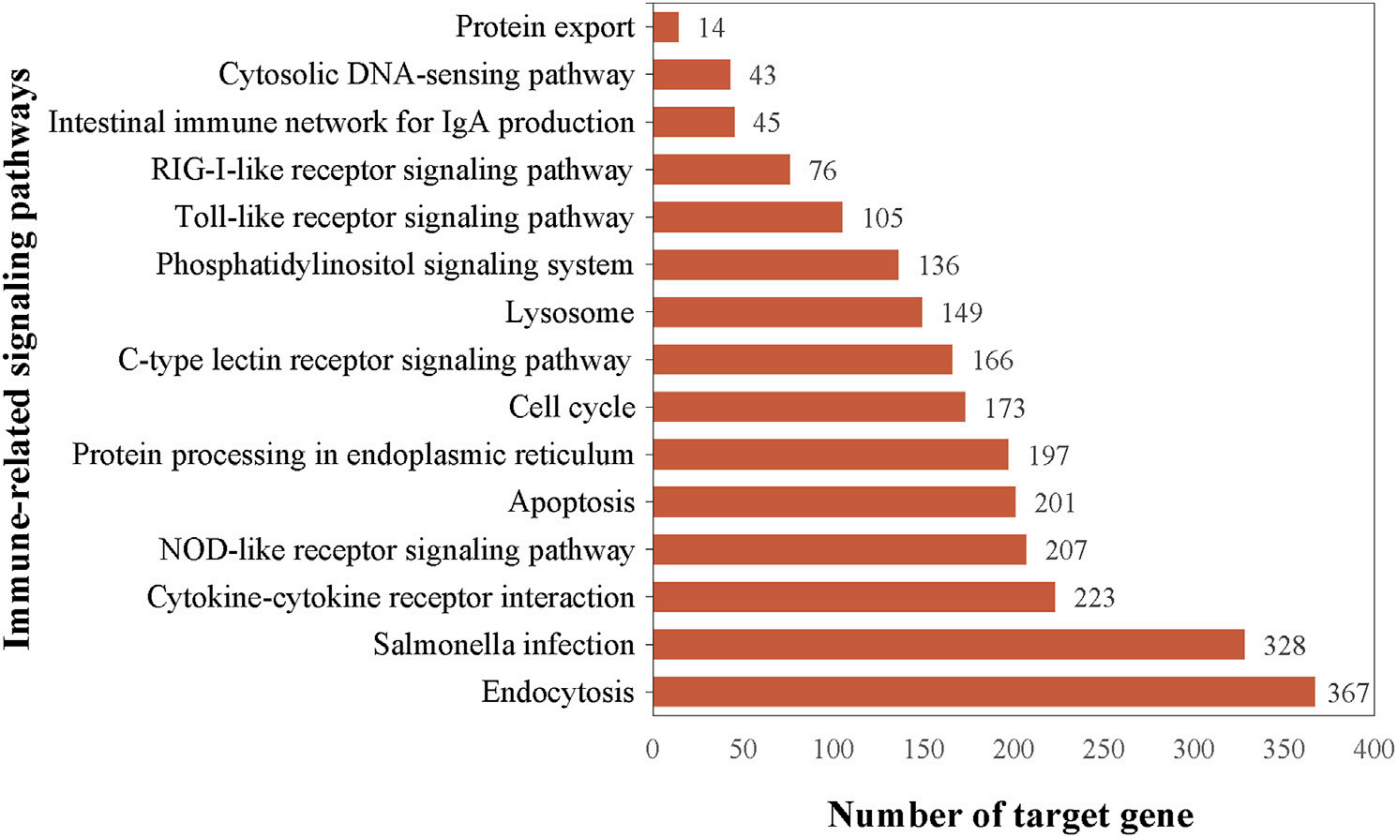
Volcano plot of the number of differentially expressed miRNAs between control and AF treated samples.

Candidate miRNAs in the two groups were screened for differentially expressed miRNAs(DEMs) according to fold difference in expression (|fold change| > 2) and significance of expression difference (p-value <0.05). Totally, 30 DEMs were found to be significantly differentially expressed in control and AF treatment samples of liver, of which 24 miRNAs were upregulated and 6 miRNAs were downregulated (Figure 2; Supplementary Table S2). These DEMs may play a vital regulatory role in the immune response during *A. frigida* therapy.

### 3.4 Validation of miRNAs by fluorescence quantitative PCR

**FIGURE 3:**
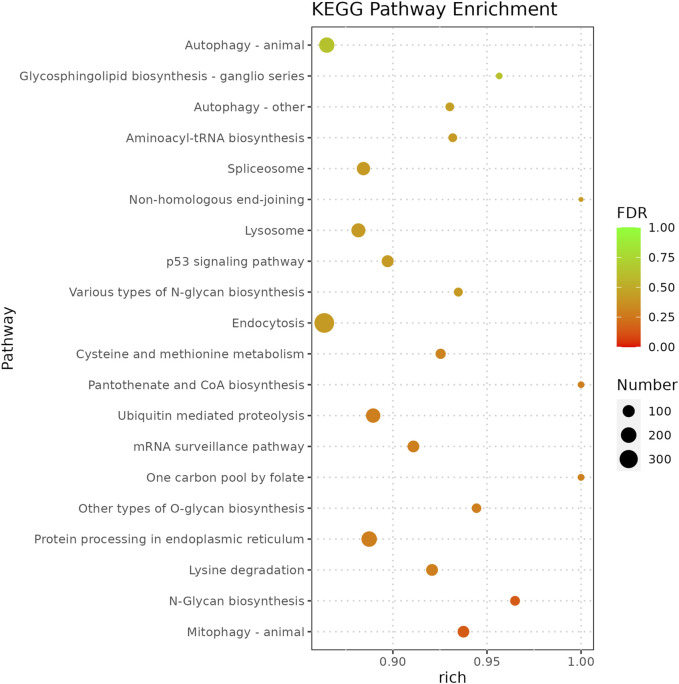
Relative expression levels of six miRNAs in control and AF samples by qRT-PCR and small RNA sequencing. Relative expression levels detected by qRT-PCR are expressed as 2^−ΔΔCt^, miRNA expression levels analysed by high-throughput sequencing are represented by statistically measured reads count values.

To make the subsequent analysis more convincing, we used qRT-PCR detection technology to verify the miRNA sequencing results. Six differentially expressed miRNAs related to immunity were selected for validation, and the results revealed that qRT-PCR and next-generation sequencing (NGS) analyses of the six miRNAs had analogous expression patterns (Figure 3). The expression of manu-mir-1-9, manu-mir-202-3, manu-mir-27-3, manu-undef-374 and manu-undef-508 was significantly upregulated after treatment with *A. frigida*, while the expression of manu-undev-411 was inhibited, indicating that they played a crucial role in response to the immune response of loach. Anyhow, the results of qRT-PCR indicated that the differentially expressed miRNAs provided by NGS analysis were plausible and realistically responded to the changes in miRNA expression profiles of loach after *A. frigida* treatment.

### 3.5 Prediction of miRNA target gene

It is universally acknowledged that miRNAs usually bind to the 3′ untranslated region of mRNAs and inhibit the translation process of target genes to function (Bagga et al., 2005; Selbach et al., 2008). Therefore, it is particularly important to use bioinformatics methods to predict the targets of miRNAs regulated by *A. frigida*. We used miRanda software for prediction and found that 157 miRNAs were forecasted, with a total number of target genes and target sites of 73312 and 894895, respectively. The fact that each miRNA can predict multiple target mRNAs at the same time and each target gene may have multiple target sites, which demonstrates the complexity of the miRNA-mRNA interaction network.

### 3.6 Functional annotation and analysis of miRNA

**FIGURE 4:**
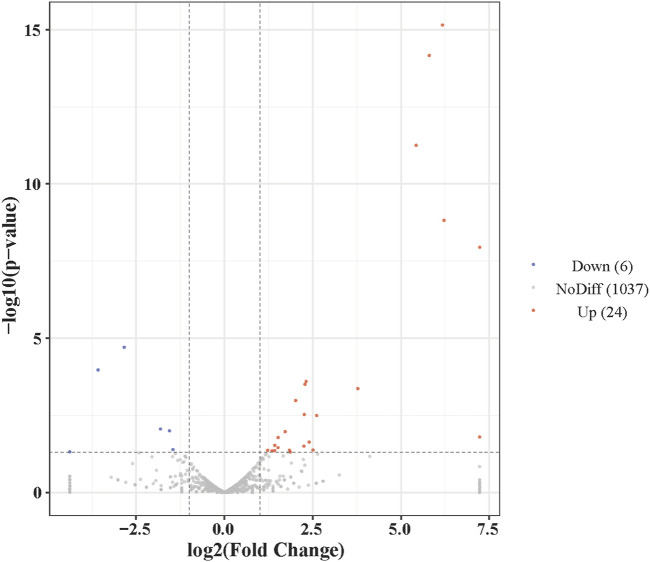
Gene ontology (GO) analysis was performed on the predicted target genes of differentially expressed miRNAs in the control and AF treated samples.

**FIGURE 5:**
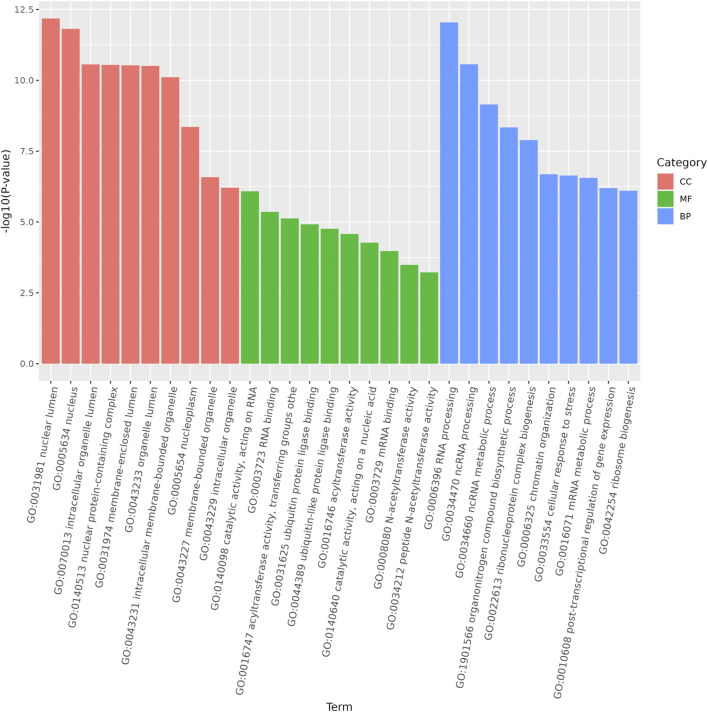
Immune-related signaling pathways enriched by target genes regulated by differentially expressed miRNAs and their targets distribution.

**FIGURE 6:**
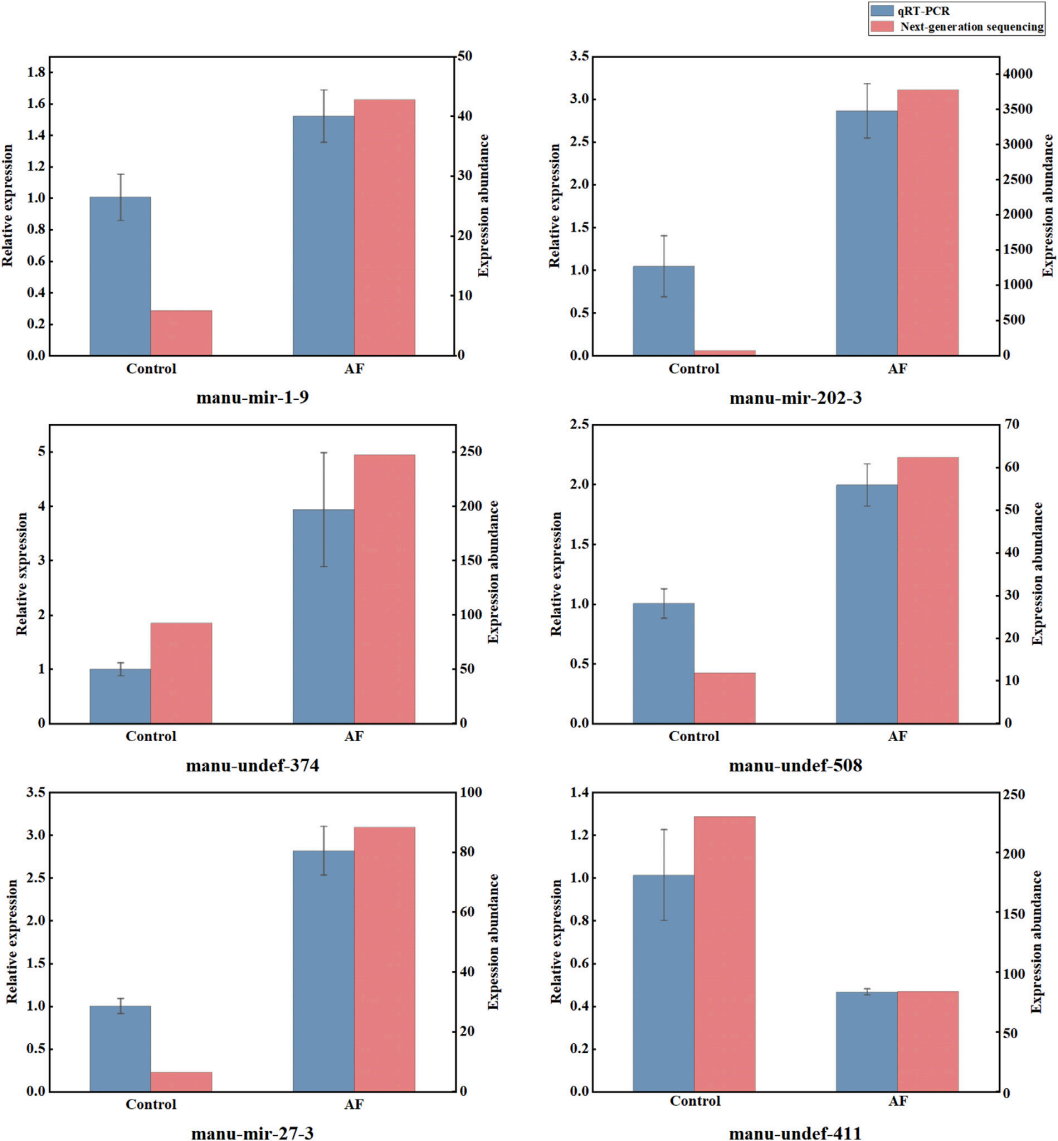
Top 20 enriched KEGG pathways enriched for target genes differentially expressing mi RNA in control and AF treated samples.

Functional analysis of the predicted targets of differential miRNAs facilitates the understanding of physiological processes regulated by miRNAs. In GO enrichment analyses, the target genes were clustered into three categories, biological processes, cellular components, and molecular functions, and due to the large number of GO terms enriched in each of these categories, Figure 4 demonstrates only the top 10 most significantly enriched GO terms in each of the GO categories. Within the category of cellular components, target genes are predominantly enriched in the nuclear fraction, organelles, protein complexes, and luminal compartments. In addition, the molecular function consists mainly of GO terms such as catalytic activity, binding activity, and transferase activity. Biological processes entries are primarily concerned with cellular processing, synthesis and metabolism, innate immune response, and transcriptional regulation. The results of the above analyses give us a preliminary understanding of the biological functions played by miRNAs regulated by *A. frigida*. Further pathway enrichment analysis of all target genes using KEGG database, we found that a total of 15 pathways related to immune response were enriched (Figure 5), which included apoptosis, lysosome, *salmonella* infection, toll-like receptor signaling pathway, NOD-like receptor signaling pathway, and so on, and these pathways may play key roles in immune regulation in loach. Additionally, there were 30 significantly enriched pathways in the KEGG enrichment analysis results (Figure 6), which belonged to four broad categories, with the highest percentage of signalling pathways enriched for metabolism. Figure 6 shows that the target genes of differentially expressed miRNAs are mainly involved in pathways such as amino acid metabolism, lipid metabolism, glycan synthesis and metabolism, signal transduction and immune system. The results suggest that differential miRNAs regulate the antimicrobial immune response in loach by affecting these signalling pathways.

In the present study, high-throughput sequencing results showed that the addition of *A. frigida* to the feed induced changes in the expression of loach miRNAs, and that these differentially expressed miRNAs regulate a number of immune-related target genes, which participate in a variety of innate and adaptive immune pathways (Figure 5). We found 201 and 367 target genes enriched to the autophagy and endocytosis pathways, respectively, both accounting for more than 80% of the total number of genes within the pathways. Autophagy and endocytosis pathways are the first line of defence against invasion by foreign pathogens in fish and play an important role in innate immunity (Cui et al., 2019). It has been shown that viral or bacterial infections can significantly enhance autophagic activity in fish. For example, upregulation of novel _ miR - 61, novel _ miR - 205, and novel _ miR - 320 expression associated with autophagic pathways was found in spleens of ISKNV-infected *Siniperca chuatsi* (Zhou et al., 2022b). Moreover, in miRNA immunity studies in fish, RIG-I-like receptor signaling pathway (RLRs), Toll-like receptor signaling pathway (TLRs), NOD-like receptor signaling pathway (NLRs) are key pathways for miRNAs to exert antimicrobial and immune effects. Such as Xu et al. found that differentially expressed miRNAs induced pro-inflammatory cytokine expression by negatively regulating the TLR signalling pathway in miiuy croaker infected with *Vibrio anguillarum* (Xu et al., 2016). Sun et al. reported that miR-210 can target DUBA inhibition in response to poly(I:C) stimulation, thereby indirectly activating the RLRs signalling pathway and regulating the immune response of the organism (Sun et al., 2018). In our study, the target genes of differentially expressed miRNAs were also significantly enriched in the three types of receptor signalling pathways, with the highest number of genes annotated in the NLRs signalling pathway, which has a similar conclusion to the above study. Therefore, it is reasonable to believe that the extract of *A. frigida* is able to modulate the expression of miRNAs in loach, thereby mediating the immune response-related pathway to exert antimicrobial activity. Pre-feeding of experimental loach with the extract of *A. frigida* enhances immunoprotection to the extent that natural and adaptive immune responses are rapidly initiated in defence against invasion by exogenous pathogenic bacteria. The results of this study provide an effective basis for the development of plant-derived antibiotics in the antimicrobial therapeutic applications of aquaculture in future. Although we have identified differentially expressed miRNAs and changes in their expression profiles in loach, and also performed miRNA target gene prediction, the mechanism of the specific biological functions exerted by miRNAs has yet to be thoroughly investigated.

## 4 Conclusion

To summarize, we analysed the miRNA profiles of loach treated with the Chinese herb *A. frigida* and then infested with *Aeromonas hydrophila* at the genomic level by Illumina sequencing for the first time, and successfully identified 30 significantly differentially expressed miRNAs, which may play an important role in the regulation of the antibacterial immune response of loach. Upon functional enrichment analysis, the target genes regulated by these differentially expressed miRNAs were found to be involved in innate immune response-related pathways such as lysosomes, endocytosis, apoptosis, cytokine-cytokine receptor interaction, phosphatidylinositol signaling system, protein processing in endoplasmic reticulum, protein export and others. In addition, some pathways associated with the antibacterial immune response such as RLR signaling pathway, TLR signaling pathway and NLR signaling pathway were also significantly enriched by many differential miRNAs. Then, due to the complexity of the miRNA-mRNA co-expression network, the specific mechanisms of antibacterial immunity regulated by loach miRNAs need to be thoroughly investigated. In conclusion, our results that cast a new light on the role of miRNAs in the immune system of loach and provide new ideas for the application of *A. frigida* in the disease control of loach. Looking forward, further attempts of the functions and modes of action of fish miRNAs could prove quite beneficial to the healthy and sustainable development of fisheries.

## Data Availability

The datasets presented in this study can be found in online repositories. The names of the repository/repositories and accession number(s) can be found below: https://www.ncbi.nlm.nih.gov/genbank/, SRR29790699.
