# Supplementary material for: Potential immunomodulatory effects of the extract from Artemisia frigida Willd on loaches infested with Aeromonas hydrophila revealed by microRNA analysis
Source: Front Genet. 2025 Apr 15;16:1584539. doi: 10.3389/fgene.2025.1584539 (PMC12037634; doi:10.3389/fgene.2025.1584539)
Supplement: Supplementary file 1 [file DataSheet1.docx]

**Additional file 1** Classification statistics of ncRNA reads

| Type | Control | AF |
| --- | --- | --- |
| rRNA | 72221 | 55247 |
| snoRNA | 4324 | 3901 |
| snRNA | 2577 | 2292 |
| tRNA | 15154 | 11338 |
| Total ncRNA | 94276 | 72778 |

**Additional file 2** Differentially expressed miRNAs in the two samples

| miRNA id | Control | Treatment | Log2FC | Pval | Regulation |
| --- | --- | --- | --- | --- | --- |
| manu-mir-1-1 | 44.06137483 | 211.2280889 | 2.261215279 | 0.002986 | Up Regulation |
| manu-mir-1-5 | 19.34401822 | 118.1760674 | 2.610978482 | 0.003224 | Up Regulation |
| manu-mir-1-9 | 7.522673752 | 42.80392991 | 2.50842583 | 0.042135 | Up Regulation |
| manu-mir-202-3 | 67.70406376 | 3777.912075 | 5.802202885 | 6.86E-15 | Up Regulation |
| manu-mir-27-3 | 6.448006073 | 88.39942047 | 3.777111904 | 0.000432 | Up Regulation |
| manu-mir-31-1 | 447.0617544 | 2174.625743 | 2.282221097 | 0.000314 | Up Regulation |
| manu-mir-460-4 | 0 | 21.40196495 | Inf | 0.016005 | Up Regulation |
| manu-mir-728-1 | 20.4186859 | 74.44161723 | 1.866219378 | 0.049261 | Up Regulation |
| manu-undef-1019 | 12.89601215 | 61.41433422 | 2.251650415 | 0.031673 | Up Regulation |
| manu-undef-162 | 42.98670715 | 3112.590121 | 6.178081197 | 7.06E-16 | Up Regulation |
| manu-undef-204 | 3.224003036 | 240.0742156 | 6.218483551 | 1.52E-09 | Up Regulation |
| manu-undef-30 | 20.4186859 | 879.3416036 | 5.428461802 | 5.61E-12 | Up Regulation |
| manu-undef-374 | 92.42142038 | 247.5183773 | 1.421236477 | 0.044013 | Up Regulation |
| manu-undef-385 | 126.8107861 | 417.8035767 | 1.720147382 | 0.010724 | Up Regulation |
| manu-undef-407 | 1.074667679 | 160.9799973 | 7.226847024 | 1.13E-08 | Up Regulation |
| manu-undef-462 | 2430.898289 | 9843.042839 | 2.017614842 | 0.001048 | Up Regulation |
| manu-undef-508 | 11.82134447 | 62.34485443 | 2.398876368 | 0.023272 | Up Regulation |
| manu-undef-559 | 181.6188377 | 487.5925929 | 1.424762361 | 0.029906 | Up Regulation |
| manu-undef-613 | 514.7658181 | 2558.000072 | 2.313028142 | 0.000252 | Up Regulation |
| manu-undef-658 | 77.37607287 | 221.4638113 | 1.517111558 | 0.035388 | Up Regulation |
| manu-undef-797 | 298.7576147 | 857.9396386 | 1.521900664 | 0.016609 | Up Regulation |
| manu-undef-834 | 140.7814659 | 358.2502829 | 1.34751043 | 0.045116 | Up Regulation |
| manu-undef-879 | 23.64268893 | 84.6773396 | 1.840581818 | 0.043313 | Up Regulation |
| manu-undef-9 | 1422.860007 | 3327.54029 | 1.225662411 | 0.043374 | Up Regulation |
| manu-undef-149 | 19.34401822 | 0.930520215 | -4.377706205 | 0.048352 | Down Regulation |
| manu-undef-411 | 229.9788833 | 83.74681939 | -1.457395094 | 0.040881 | Down Regulation |
| manu-undef-412 | 133.2587922 | 11.16624259 | -3.577015013 | 0.000107 | Down Regulation |
| manu-undef-475 | 1064.99567 | 148.8832345 | -2.838594356 | 1.98E-05 | Down Regulation |
| manu-undef-511 | 336.3709835 | 95.84358219 | -1.811299524 | 0.008795 | Down Regulation |
| manu-undef-662 | 30773.10898 | 10468.35242 | -1.555635806 | 0.010106 | Down Regulation |
